# Validating Orthopaedic Data Evaluation Panel (ODEP) Ratings Across 9 Orthopaedic Registries: Total Hip Implants with an ODEP Rating Perform Better Than Those without an ODEP Rating

**DOI:** 10.2106/JBJS.23.00793

**Published:** 2024-05-31

**Authors:** Lotje A. Hoogervorst, Maartje M. van Tilburg, Anne Lübbeke, Tim Wilton, Rob G.H.H. Nelissen, Perla J. Marang-van de Mheen

**Affiliations:** 1 Department of Orthopaedics, Leiden University Medical Center, Leiden, The Netherlands; 2 Department of Biomedical Data Sciences, Medical Decision Making, Leiden University Medical Center, Leiden, The Netherlands; 3 Division of Orthopaedic Surgery and Traumatology, Geneva University Hospitals, Geneva Switzerland; 4 University of Geneva, Geneva, Switzerland; 5 Nuffield Department of Orthopaedics, Rheumatology and Musculoskeletal Sciences, University of Oxford, Oxford, United Kingdom; 6 Department of Orthopaedics, Derby Teaching Hospitals NHS Foundation Trust, Derby, United Kingdom; 7 Orthopaedic Data Evaluation Panel, United Kingdom; 8 Beyond Compliance Steering Committee, Halesowen, United Kingdom; 9 Safety & Security Science, Centre for Safety in Healthcare, Delft University of Technology, Delft, The Netherlands

## Abstract

**Background:**

Orthopaedic Data Evaluation Panel (ODEP) ratings of total hip (TH) and total knee (TK) implants are informative for assessing implant performance. However, the validity of ODEP ratings across multiple registries is unknown. Therefore, we aimed to assess, across multiple registries, whether TH and TK implants with a higher ODEP rating (i.e., an A* rating) have lower cumulative revision risks (CRRs) than those with a lower ODEP rating (i.e., an A rating) and the extent to which A* and A-rated implants would be A*-rated on the basis of the pooled registries’ CRR.

**Methods:**

Implant-specific CRRs at 3, 5, and 10 years that were reported by registries were matched to ODEP ratings on the basis of the implant name. A meta-analysis with random-effects models was utilized for pooling the CRRs. ODEP benchmark criteria were utilized to classify these pooled CRRs.

**Results:**

A total of 313 TH cups (54%), 356 TH stems (58%), 218 TH cup-stem combinations (34%), and 68 TK implants (13%) with unique brand names reported by registries were matched to an ODEP rating. Given the low percentage that matched, TK implants were not further analyzed. ODEP-matched TH implants had lower CRRs than TH implants without an ODEP rating at all follow-up time points, although the difference for TH stems was not significant at 5 years. No overall differences in CRRs were found between A* and A-rated TH implants, with the exception of TH cup-stem combinations, which demonstrated a significantly lower CRR for A*A*-rated cup-stem combinations at the 3-year time point. Thirty-nine percent of A*-rated cups and 42% of A*-rated stems would receive an A* rating on the basis of the pooled registries’ CRR at 3 years; however, 24% of A-rated cups and 31% of A-rated stems would also receive an A* rating, with similar findings demonstrated at longer follow-up.

**Conclusions:**

At all follow-up time points, ODEP-matched TH implants had lower CRRs than TH implants without an ODEP rating. Given that the performance of TH implants varied across countries, registries should first validate ODEP ratings with use of country-specific revision data to better guide implant selection in their country. Data source transparency and the use of revision data from multiple registries would strengthen the ODEP benchmarks.

**Level of Evidence:**

Therapeutic Level III. See Instructions for Authors for a complete description of levels of evidence.

In the United States, medical devices are regulated by the Food and Drug Administration^1^. In the European Union, medical devices are regulated according to the Medical Device Regulation (MDR), which aims to provide “a robust, transparent, predictable and sustainable regulatory framework for medical devices which ensures a high level of safety and health whilst supporting innovation.”^2,3^ To ensure patient safety, the MDR requires manufacturers to monitor the performance of their implants, including total hip (TH) and total knee (TK) implants, with use of benchmarking—that is, “a systematic process of determining whether an implant meets specified performance levels.”^4,5^ Several methods for benchmarking TH and TK implants are utilized. These methods include comparing the performance of an implant to that of the best-performing implant, comparing it to the average performance of comparable implants, or comparing it to absolute thresholds determined by objective performance criteria (OPC)^6-15^.

An example of the use of OPC to promote the evidence-based selection of implants is the Orthopaedic Data Evaluation Panel (ODEP) rating, which is assigned to implants that show .evidence of meeting survivorship criteria^10^. ODEP ratings are available for TH components (cups and stems), TK implants (tibiofemoral combinations), unicondylar knee implants, shoulder components (glenoids and stems), reverse shoulder implants, total elbow implants, and spine implants (cervical discs). Implants are benchmarked by ODEP on the basis of revision data from observational studies (e.g., single-center studies, manufacturers’ in-house sources, and registry data). Thus, not all ODEP ratings are based on registry data. The submitted data are supplied by manufacturers with use of standardized ODEP submission forms^16^. Not all implants on the market are submitted to ODEP since data submission is voluntary, but surgeons and hospitals are encouraged to use ODEP-rated implants. As different data sources can be utilized by manufacturers to submit their application for an ODEP rating, the data may not be representative of daily clinical practice. Therefore, before submission, manufacturers have to declare that the submitted clinical data are “representative of the results of all studies conducted in relation to it.”^17^ The ODEP rating includes a number (representing the years of evidence) and a letter (representing the strength of the evidence). The latter denotes the performance of implants at specific time points (i.e., 3, 5, 7, 10, 13, and 15 years) based on the OPC, which include the minimum number of centers and surgeons, size of the cohort, number of patients at risk, and the maximum revision rate. Implants can be rated as A* (highest rating), A (lower rating), or B (a rating that is assigned either to implants that are extremely important but have limited usage or to new implants that are introduced in a limited manner), starting from 3 years of evidence. Implants that do not meet the ODEP benchmark criteria (Table I) are not rated. Although originally developed for use in the United Kingdom (U.K.), the ODEP rating is increasingly utilized internationally for the quality assessment of implants^18-20^. In the Dutch Arthroplasty Register, 100% of all TH cups and TH stems and 92% of all TK implants utilized in 2019 were assigned an ODEP rating. In the U.K., comparable numbers were reported in 2018^19,21^. Although ODEP ratings are increasingly utilized, to our knowledge, an external validation of ODEP ratings across multiple registries has never been undertaken.

**TABLE I:** ODEP Benchmark Criteria for TH and TK Implants^42^,^43^†‡

TH Implant: Criteria for A* Ratings	3A*	5A*	7A*	10A*	13A*	15A*
Minimum no. of centers outside development center(s)	3	3	3	3	3	3
Minimum no. of surgeons outside development center(s)	3	3	3	3	3	3
Minimum total cohort	150	250	350	500	500	500
Minimum at risk at benchmark time	150	225	300	400	400	400
Maximum revision rate§	3.0%	3.5%	4.0%	5.0%	6.5%	8.0%

TH Implant: Criteria for A Ratings	3A	5A	7A	10A	13A	15A
Minimum no. of centers and surgeons	3	3	3	3	3	3
Minimum total cohort	150	250	350	500	500	500
Minimum at risk at benchmark time	72	66	60	51	42	40
Maximum revision rate§	5.0%	5.5%	6.0%	7.0%	8.5%	10.0%

TH Implant: Criteria for B Ratings	3B	5B	7B	10B	13B	15B
Minimum no. of centers and surgeons	1	1	1	1	1	1
Minimum total cohort	100	100	100	100	100	100
Minimum at risk at benchmark time	40	40	40	40	40	40
Maximum value of 95% lower confidence limit for revision rate	3.0%	3.5%	4.0%	5.0%	6.5%	8.0%

TK Implant: Criteria for A* Ratings	3A*	5A*	7A*	10A*	13A*	15A*
Minimum no. of centers outside development center(s)	3	3	3	3	3	3
Minimum no. of surgeons outside development center(s)	3	3	3	3	3	3
Minimum total cohort	150	250	350	500	500	500
Minimum at risk at benchmark time	150	225	300	400	400	400
Maximum revision rate§	3.5%	4.0%	4.5%	5.0%	6.0%	6.5%

TK Implant: Criteria for A Ratings	3A	5A	7A	10A	13A	15A
Minimum no. of centers and surgeons	3	3	3	3	3	3
Minimum total cohort	150	250	350	500	500	500
Minimum at risk at benchmark time	66	60	55	51	51	45
Maximum revision rate§	5.5%	6.0%	6.5%	7.0%	8.0%	8.5%

TK Implant: Criteria for B Ratings	3B	5B	7B	10B	13B	15B
Minimum no. of centers and surgeons	1	1	1	1	1	1
Minimum total cohort	100	100	100	100	100	100
Minimum at risk at benchmark time	66	60	55	51	45	42
Maximum value of 95% lower confidence limit for revision rate	3.5%	4.0%	4.5%	5.0%	6.0%	6.5%

† Reproduced, with modification, from: Orthopaedic Data Evaluation Panel (ODEP). ODEP Hip Criteria Table and ODEP Knee Criteria Table. www.odep.org.uk. Reproduced with permission.

‡ For TH and TK implants, the criteria for a pre-entry A* rating is the launch of the product under Beyond Compliance, and the criteria for a pre-entry A rating is the supplying of the product details to ODEP.

§ The upper 95% CI bound for the Kaplan-Meier revision rate (1 minus survival) must be lower than the specified level.

Therefore, we aimed to assess, across multiple registries, whether TH and TK implants with a higher ODEP rating (i.e., an A* rating) have lower cumulative revision risks (CRRs) than those with a lower ODEP rating (i.e., an A rating) and the extent to which A* and A-rated implants would receive the A* rating on the basis of the pooled registries’ CRR. Since the maximum revision rate for an A* rating is lower than that for an A rating, we hypothesized that A*-rated implants would have lower CRRs across the registries than A-rated implants. Furthermore, we expected that the majority—but not all—of the A*-rated implants would also be A*-rated on the basis of the pooled registries’ CRR, as revision risks are also influenced by variables such as surgeon factors that potentially affect implant performance.

## Materials and Methods

### The ODEP Rating

The data submitted to ODEP is evaluated by a voluntary, independent panel of orthopaedic experts. To prevent camouflage (i.e., when the performance of a specific implant-design variant is concealed as a result of different variants existing under the same implant name)^22^, the panel reviews implants at the product-code level^22^ (Table I^10^). After being assigned an ODEP rating, manufacturers have to resubmit new evidence at every ODEP milestone to prevent their implant ratings from being lapsed, which some manufacturers may not do^10^. ODEP usually provides a grace period of 1 year before lapsing an ODEP rating. Implants that do not meet the benchmark criteria do not receive an ODEP rating.

### Matching Registry Data to ODEP Ratings

European registries were identified in a previous systematic review and were supplemented with non-European registries listed by the Australian Orthopaedic Association National Joint Replacement Registry (AOANJRR)^23,24^. Registries were included if they reported implant-specific CRRs with standard errors (SEs) and/or 95% confidence intervals (CIs) to allow the pooling of data and if they were “active” (i.e., “published at least one annual report and/or peer-reviewed paper containing registries’ data, during or later than 2018”^24^). The CRR was defined as the number of patients who needed to undergo a revision up to a certain time point as a proportion of the total number of patients who were at risk after a primary procedure.

For TH components (cups or stems), TH cup-stem combinations, and TK implants (tibiofemoral combinations), the following registry data were extracted: name, manufacturer, type of fixation, number of implants, and the CRR with the SE and/or 95% CI. If only the 95% CI was provided, then the SE was calculated by subtracting the values of the upper and lower bounds of the 95% CI and dividing the result by 3.92^25^.

**Fig. 1:**
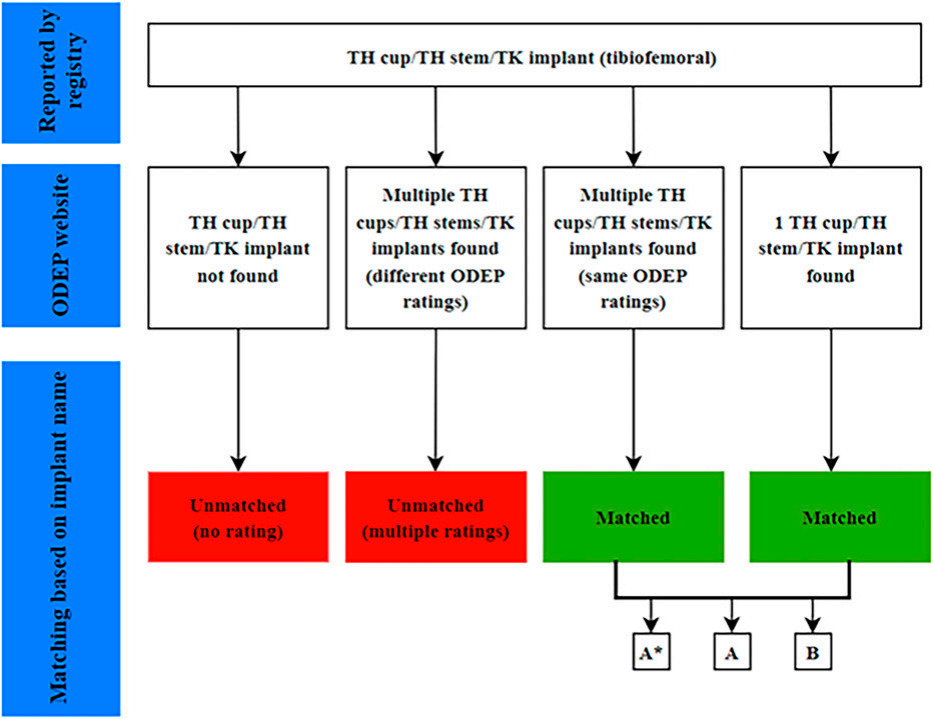
Diagram showing the process for matching registry-reported TH cups, TH stems, and TK implants to the ODEP rating for that implant.

**Fig. 2:**
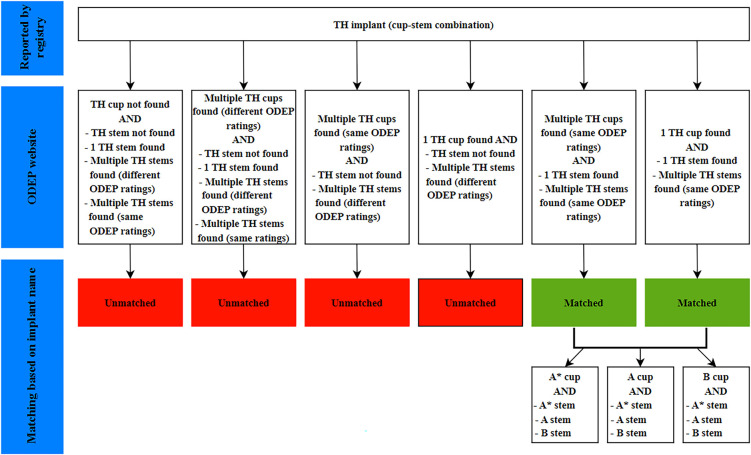
Flowchart showing the process for matching registry-reported TH implants (cup-stem combinations) to the ODEP rating for that implant.

The implants in the registry data were identified, on the basis of the implant name, as having received an ODEP rating or not (Figs. 1 and 2). ODEP-matched implants with a B rating were excluded because such a rating is assigned for implants with limited usage.

### Statistical Analysis

Before comparing higher and lower-rated implants with respect to their CRRs, we assessed whether ODEP-rated implants represented a selected group of implants. Therefore, with use of independent t tests, we evaluated whether ODEP-matched implants differed from unmatched implants with and without multiple ODEP ratings (red boxes; Figs. 1 and 2) in terms of the CRR.

For ODEP-matched implants, random-effects models were utilized to calculate the pooled registries’ CRR at 3, 5, and 10 years for A*- and A-rated implants. These models included the DerSimonian-Laird estimator to consider the extent of heterogeneity among the implant designs^26^. The ODEP rating (A* or A) was included as a factor to test for group differences. This analysis was performed separately for TH components and TK implants. Similar random-effects models were utilized to compare A*A*-rated and AA-rated TH cup-stem combinations. The I^2^ was utilized to estimate the extent of heterogeneity in the pooled registries’ CRR, which was defined as low (25%), moderate (50%), or high (75%)^27,28^. To explore possible reasons for the observed heterogeneities, TH components, TK implants, and TH cup-stem combinations were stratified by fixation type and the analyses were repeated. Additionally, another analysis was performed with TH cup-stem combinations stratified by whether the individual components were from the same manufacturer or different manufacturers.

To answer the second research question, random-effects models were utilized to calculate the pooled CRR with 95% CI at 3, 5, and 10 years for each TH component across all registries in which the component was reported. The pooled CRR was then compared with ODEP benchmark criteria (Table I) to assess whether the TH component met the criteria for an A* rating. We then calculated the percentage of A*-rated TH components that would receive an A* rating on the basis of the pooled registries’ CRR and performed a similar calculation for A-rated TH components. Considering that the performance of an implant may differ across registries, we also examined the median number (and range) of registries in which each TH component would be assigned an A* rating as well as examined how many TH components would consistently get an A* rating in all registries in which the component was reported.

The metafor package in R (R Foundation for Statistical Computing; version 4.1.2) was utilized for meta-analyses. The level of significance was set at p < 0.05.

## Results

**Fig. 3:**
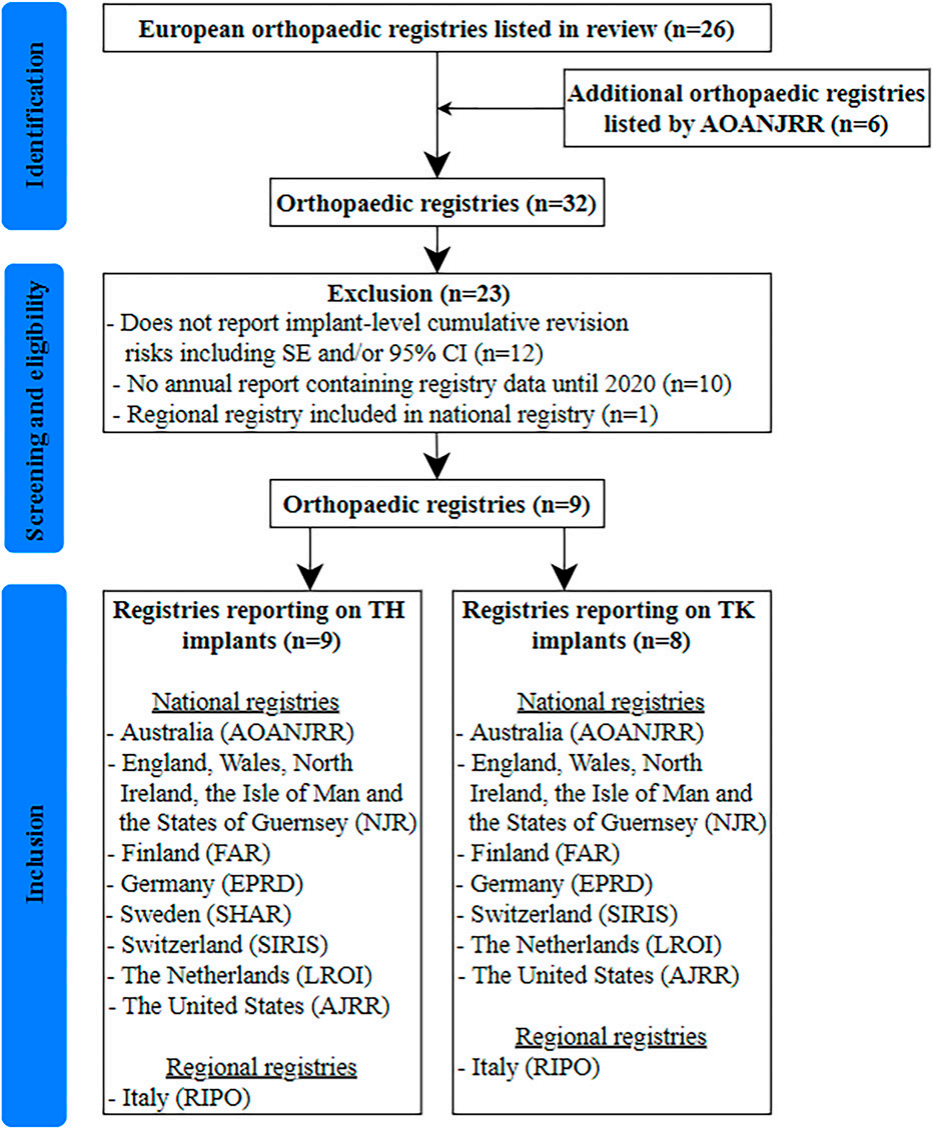
Flowchart showing the selection process for registries. AOANJRR = Australian Orthopaedic Association National Joint Replacement Registry, NJR = National Joint Registry, FAR = Finnish Arthroplasty Register, EPRD = The German Arthroplasty Registry, SHAR = Swedish Hip Arthroplasty Register, SIRIS = Swiss National Hip & Knee Joint Registry, LROI = Dutch Arthroplasty Register, AJRR = American Joint Replacement Registry, RIPO = Register of Orthopaedic Prosthetic Implantology.

Nine registries were included (Fig. 3). The latest annual reports of 8 registries^19,29-35^, consisting of data up to December 2019, and the up-to-date data (as of March 2021) from the website of 1 registry^36^ were utilized. The mean percentage completeness of patient or procedure-level data in the included registries was 87.3% (range, 40%^29^ to 99%^19^).

Nine registries reported on a total of 583 TH cups with unique brand names (2,615,890 implants) and 618 TH stems (2,567,442 implants), and 8 registries reported on a total of 634 TH cup-stem combinations (2,266,864 implants) and 508 TK implants (2,940,899 implants) (see Appendix Supplementary Tables 1 through 4). A total of 313 (54%) of the unique TH cups, 356 (58%) of the unique TH stems, 218 (34%) of the unique TH cup-stem combinations, and 68 (13%) of the unique TK implants that were reported by registries were matched to an ODEP rating. The percentage of implants with a matched ODEP rating varied widely between registries, ranging from 35% to 69% of cups, 46% to 80% of stems, 22% to 55% of TH cup-stem combinations, and 6% to 20% of TK implants. For implants that were unmatched as a result of multiple ODEP ratings, the median number of possible ODEP ratings was 2 (range, 2 to 6) for cups, 2 (range, 2 to 8) for stems, and 4 (range, 2 to 48) for TK implants (data not shown). Since only 13% of TK implants were matched, they were not further analyzed. The failure to match most of the TK implants was primarily due to the fact that the granularity with which ODEP ratings are applied to a TK implant is much more detailed than most registry reports of a TK implant.

### ODEP-Matched Versus ODEP-Unmatched TH Implants

ODEP-matched cups had significantly lower 3, 5, and 10-year CRRs than unmatched cups without an ODEP rating, and ODEP-matched stems had significantly lower 5 and 10-year CRRs than unmatched stems without an ODEP rating. However, ODEP-matched cups and stems had comparable CRRs to unmatched cups and stems with multiple ODEP ratings (Table II). ODEP-matched TH cup-stem combinations had significantly lower CRRs than unmatched TH cup-stem combinations at all follow-up points (Table III).

**TABLE II:** Cumulative Revision Risks of ODEP-Matched Versus ODEP-Unmatched Components

	Matched Components		Unmatched Components with Multiple ODEP Ratings		Matched Components Versus Unmatched Components with Multiple ODEP Ratings	Unmatched Components with No ODEP Rating		Matched Components Versus Unmatched Components with No ODEP Rating
	Revision Risk	N	Revision Risk	N	Mean Difference (95% CI)	Revision Risk	N	Mean Difference (95% CI)
Cups								
3 years	2.6%	1,270,520	2.5%	645,191	0.1% (−0.25, 0.39)	3.2%	379,345	**−0.6% (−0.94, −0.32)†**
5 years	3.1%	1,406,957	3.2%	631,813	−0.1% (−0.49, 0.30)	5.1%	370,942	**−2.0% (−2.58, −1.37)†**
10 years	5.6%	944,820	5.4%	506,671	0.2% (−0.79, 1.11)	11.8%	196,116	**−6.3% (−8.09, −4.43)†**
Stems								
3 years	2.7%	1,423,161	2.7%	165,456	0.0% (−0.47, 0.46)	2.9%	692,944	−0.2% (−0.46, 0.09)
5 years	3.4%	1,418,673	3.4%	162,655	0.0% (−0.82, 0.82)	4.2%	675,774	**−0.7% (−1.30, −0.16)‡**
10 years	6.7%	1,004,520	5.7%	112,264	1.0% (−1.73, 3.80)	8.8%	606,571	**−2.0% (−3.74, −0.33)§**

† P < 0.001.

‡ P = 0.013.

§ P = 0.019.

**TABLE III:** Cumulative Revision Risks of ODEP-Matched Versus ODEP-Unmatched TH Cup-Stem Combinations

	Matched TH Cup-Stem Implants		Unmatched TH Cup-Stem Implants		Matched Versus Unmatched Implants
	Revision Risk	N	Revision Risk	N	Mean Difference (95% CI)
3 years	2.6%	799,382	2.9%	1,405,493	**−0.3% (−0.58, −0.08)†**
5 years	3.0%	793,761	4.0%	1,365,984	**−1.0% (−1.52, −0.47)‡**
10 years	5.2%	503,730	8.6%	1,006,928	**−3.4% (−5.08, −1.66)‡**

† P = 0.010.

‡ P < 0.001.

### A*-Rated Versus A-Rated TH Implants

No overall differences in CRRs were found between A*-rated and A-rated TH implants, with the exception of the CRRs for TH cup-stem combinations, which were significantly lower for A*A*-rated cup-stem combinations at the 3-year time point (Tables IV and V). Moderate to high (range, 64% to 95%) heterogeneity was found, reflecting variation in CRRs between implants (Tables IV and V). To explore this heterogeneity, the analyses were repeated with the implants stratified by fixation type, which again showed no significant differences in the CRRs at 3, 5, and 10 years for all analyzed groups and demonstrated moderate to high heterogeneity (data not shown). Among TH cup-stem combinations that consisted of components from the same manufacturer, A*A*-rated implants had significantly lower 3 and 5-year CRRs than AA-rated implants. Among TH cup-stem combinations with components from different manufacturers, no significant differences were found (data not shown).

**TABLE IV:** Cumulative Revision Risks of A*-Rated Versus A-Rated TH Components

	A* Components			A Components			A* Versus A Components	I^2^
	Revision Risk	N	No. of Registries Included	Revision Risk	N	No. of Registries Included	Mean Difference (95% CI)	
Cups								
3 years	2.3%	1,058,495	7	2.6%	153,979	5	−0.2% (−1.19, 0.71)	78%
5 years	2.7%	1,302,734	9	2.9%	180,830	7	−0.3% (−1.34, 0.78)	86%
10 years	4.3%	1,030,923	6	5.9%	137,499	5	−1.5% (−3.55, 0.49)	90%
Stems								
3 years	2.3%	1,098,938	7	2.3%	288,025	7	0.1% (−0.60, 0.74)	67%
5 years	3.0%	1,109,707	8	3.0%	311,695	8	0.0% (−0.76, 0.81)	70%
10 years	5.4%	1,001,275	6	6.7%	170,134	5	−1.3% (−4.63, 2.08)	95%

**TABLE V:** Cumulative Revision Risks of Higher Versus Lower-Rated TH Cup-Stem Combinations

	A*A* Cup-Stem Implants			AA Cup-Stem Implants			A* Cup + A Stem Implants			A Cup + A* Stem Implants			A*A* Versus AA Implants	
	Revision Risk	N	No. of Registries Included	Revision Risk	N	No. of Registries Included	Revision Risk	N	No. of Registries Included	Revision Risk	N	No. of Registries Included	Mean Difference (95% CI)	I^2^
3 years	2.1%	448,940	7	3.2%	16,066	4	2.5%	191,696	7	2.2%	86,761	5	**−1.1% (−0.08, −2.11)†**	66%
5 years	2.7%	452,788	8	3.7%	17,121	5	3.0%	211,212	8	2.6%	87,954	6	−1.1% (−2.15, 0.04)	64%
10 years	5.1%	351,180	5	6.0%	14,891	4	4.7%	116,519	4	4.6%	83,244	5	−0.9% (−3.45, 1.61)	79%

† P = 0.035

### ODEP Ratings Based on Pooled Registries’ CRR

Among all ODEP-matched A*-rated cups and stems, 39% of cups and 42% of stems would receive an A* rating on the basis of the pooled registries’ CRR at 3 years, 44% of cups and 35% of stems would receive such a rating at 5 years, and 30% of cups and 5% of stems would receive such a rating at 10 years (Table VI; see also Appendix Figures 1 and 2 for implant-level results). Analyzing A*-rated cups and stems that were reported by ≥2 registries resulted in similar percentages at 3 and 5 years but lower percentages at 10 years than in the previous analysis (Table VI). Cups and stems qualifying for an A* rating on the basis of the pooled registries’ CRR would receive an A* rating in a median of 1 registry at all follow-up points (range, 0 to 4 registries [cups] and 0 to 6 registries [stems]; see Appendix Table 5 and Appendix Figures 1 and 2). Three cups and 3 stems would consistently get an A* rating in all registries at 3 years; 4 cups and 2 stems, at 5 years; and 3 cups and 0 stems, at 10 years (see Appendix Tables 5 and 6).

**TABLE VI:** A*- and A-Rated TH Components That Met the OPC for an A* Rating on the Basis of Pooled Registries’ CRR

	Unique Components		Unique Components Utilized in ≥2 Registries	
	Total	No. (%) That Met the A* Benchmark	Total	No. (%) That Met the A* Benchmark
A* cups				
3 years	33	13 (39%)	23	9 (39%)
5 years	36	16 (44%)	25	11 (44%)
10 years	30	9 (30%)	18	4 (22%)
A* stems				
3 years	33	14 (42%)	25	12 (48%)
5 years	31	11 (35%)	24	8 (33%)
10 years	20	1 (5%)	14	–
A cups				
3 years	17	4 (24%)	11	3 (27%)
5 years	17	4 (24%)	11	2 (18%)
10 years	9	2 (22%)	3	1 (33%)
A stems				
3 years	29	9 (31%)	10	3 (30%)
5 years	28	9 (32%)	12	3 (25%)
10 years	13	3 (23%)	5	2 (40%)

Among all ODEP-matched A-rated cups and stems, 24% of cups and 31% of stems would receive an A* rating on the basis of the pooled registries’ CRR at 3 years, 24% of cups and 32% of stems would receive such a rating at 5 years, and 22% of cups and 23% of stems would receive such a rating at 10 years (Table VI; see also Appendix Figures 3 and 4). When analyzing A-rated cups and stems that were reported by ≥2 registries, these percentages were as follows: 27% of cups and 30% of stems at 3 years, 18% of cups and 25% of stems at 5 years, and 33% of cups and 40% of stems at 10 years (Table VI). Cups qualifying for an A* rating on the basis of the pooled registries’ CRR would receive an A* rating in a median of 0 registries at all follow-up points (range, 0 to 5 registries; see Appendix Table 7). Stems qualifying for an A* rating on the basis of the pooled registries’ CRR would receive an A* rating in a median of 1 registry (range, 0 to 2) at 3 years, 1 registry (range, 0 to 2) at 5 years, and 0 registries (range, 0 to 1) at 10 years (see Appendix Table 8). Zero cups and 1 stem would consistently receive an A* rating in all registries at 3 years; 1 cup and 2 stems, at 5 years; and 0 cups or stems, at 10 years (see Appendix Tables 7 and 8).

## Discussion

This multiregistry study showed that ODEP-matched TH implants had lower CRRs than unmatched TH implants without an ODEP rating. Among matched TH implants, CRRs did not differ between implants with a higher ODEP rating and those with a lower ODEP rating. TK implants were not analyzed because only 13% of the TK implants reported by registries were matched to an ODEP rating. Only 39% of A*-rated cups and 42% of A*-rated stems would be assigned the A* rating on the basis of the pooled registries’ CRR at 3 years. However, 24% of A-rated cups and 31% of A-rated stems would be assigned the A* rating at 3 years, with similar or lower percentages at longer follow-up. The assigned ODEP ratings varied across registries, implying that assigned ODEP ratings do not necessarily apply to the performance of TH implants in other countries. Therefore, registries should first validate ODEP ratings with use of country-specific data to better guide implant selection in their country.

In principle, OPC such as those utilized by ODEP can help stakeholders to monitor implant performance; to stimulate the continuous evaluation of implants, which may result in a higher ODEP rating and prevent losing an ODEP rating when no data are provided 2 years (for 3, 5, and 13-year ODEP ratings) or 3 years (for 7, 10, and 15-year ODEP ratings) after an ODEP rating has been assigned; and to use ODEP ratings to guide implant selection. ODEP aims to “promote evidence-based selection of implants so that patients receive the very best and safest implants.”^37^ The present study showed that ODEP-matched TH implants had better performance than unmatched TH implants without an ODEP rating, suggesting that ODEP achieves this aim by encouraging surgeons and hospitals to use ODEP-rated implants.

Some prior studies benchmarked against a predefined benchmark created by a quality institute, whereas others utilized relative benchmarks, such as the performance of the best-performing implant at that time or the average performance of similar implants^6,11-15^. Using a relative benchmark means that the judgment of whether the performance of an implant is an outlier depends on the performance of the comparator. The performance of an implant can change over time, and so too can the performance of the comparator. Therefore, even if an implant continues to have the same performance over time, that implant could become an outlier if the comparator improves. This method differs from one using absolute benchmarks such as ODEP ratings, where the OPC is predefined on the basis of what is considered to be an acceptable level of implant performance, thereby making interpretations and assessments of implant performance more straightforward^10^. However, absolute benchmarks may need to be updated over time (e.g., the ODEP rating originally had a 10-year benchmark threshold of <10%^10^), so it has to be considered whether the OPC are still acceptable.

A prerequisite for the assignment of ODEP ratings is that manufacturers must declare that the voluntarily submitted data—which may be based on various data sources—are representative of the performance of these implants in daily clinical practice^10^. The present study tested the external validity of ODEP ratings across multiple registries and showed that approximately 40% of A*-rated cups and stems would also receive the A* rating on the basis of the pooled registries’ CRR; however, we found that approximately 25% of A-rated cups and stems would receive the A* rating as well and that the A* and A ratings were inconsistent across registries. This inconsistency may be a result of differences between registries with respect to case mix; revision indications; smaller 95% CIs due to the pooling of data, which resulted in meeting the OPC; or camouflage^22^. Another explanation, particularly for implants that have been utilized for decades, and in recognition of the fact that the performance of implants has improved over time, may be that the CRR applies to patients who underwent the primary operation in a different period. For some registries, the 10-year CRR of implants may include patients who underwent the operation in the previous century, whereas for newer registries, it would include patients who underwent the operation more recently. This potential discrepancy highlights the importance of including patients from the same period when combining data across multiple registries. Nonetheless, if well-established implants continue to be utilized to the same extent, the impact of patients who underwent the operation long ago on the reported revision estimates will likely be small. This inconsistency also underscores the importance of transparent reporting of the types of submitted data sources that serve as the basis for ODEP ratings, which would allow for validation of whether the data are indeed representative, as claimed by manufacturers.

Some study limitations should be noted. First, there could have been selection bias because some implants could not be matched as a result of multiple ODEP ratings and were thus excluded. However, ODEP-matched TH cups and stems had similar CRRs to unmatched TH cups and stems with multiple ODEP ratings, making selection bias unlikely. The matching problem was due to insufficient details on implants reported by registries, resulting in a large number of compatible cup-stem combinations within 1 implant name (“camouflage”)^22^. To solve this matching problem, which was most prominent among TK implants, registries should register the product codes of implants, which is already done by a few registries^38^. Second, some registries may not have included all patients or revisions, which may have influenced the CRRs. For example, the Swedish Hip Arthroplasty Register likely underestimates revisions because it excludes revisions due to infection, and thus the actual implant-level CRRs are higher than reported^33^. These underestimated CRRs may have resulted in the assignment of an A* rating to TH implants that were commonly reported in this registry, whereas such implants might have received an A rating when including all revisions. Similarly, the American Joint Replacement Registry only includes patients with osteoarthritis who are ≥65 years old, which may again have resulted in underestimated CRRs, as the literature has generally shown a lower CRR among older patients^29,39^. Third, registries were excluded from the analysis primarily because they did not report CRRs with SEs or 95% CIs, making data comparison and pooling impossible. This highlights the importance of international agreement across registries with regard to definitions, the amount of detail (e.g., the reporting of product codes), and methodologies to enable data pooling^24^. Fourth, although we evaluated the performance of A*- and A-rated TH cup-stem combinations to give insight into possible performance differences, ODEP has never rated TH cup-stem combinations, only hip components (i.e., cups and stems) separately. Rating TH components separately is aligned with clinical practice, in which clinicians mix and match cups and stems from different manufacturers, often with excellent results^40^. However, the practice of not rating TH cup-stem combinations and instead rating TH components separately may be a potential reason for some of the differences between the ODEP ratings based on the pooled registries’ CRR and the ODEP ratings assigned by ODEP. Lastly, we only analyzed 3, 5, and 10-year CRRs because, besides the 1-year CRR, these years were the most frequently reported time points, with each registry contributing at least 2 time points. One-year CRRs were not analyzed because they are not utilized for ODEP ratings, whereas the 3-year follow-up is the first time point utilized by ODEP.

### Conclusions

In conclusion, clinicians should be encouraged to use implants with an ODEP rating, as these implants have better CRRs than unrated implants. A minority of A*-rated cups and stems would be eligible for an A* rating on the basis of the pooled registries’ CRR, with the assigned ODEP ratings varying across registries, indicating that implant performance varies across countries. Therefore, registries should first validate ODEP ratings to better guide implant selection in their country, and they should preferably do so at the product-code level to prevent camouflage. The ODEP benchmarks could be strengthened by making data submission, including transparency regarding the data source, mandatory; removing the grace period of 1 year for ODEP ratings; and using revision data from at least 2 regional, national, or multicountry registries with >95% implant-level completeness^24,41^.

## Appendix

Supporting material provided by the authors is posted with the online version of this article as a data supplement at jbjs.org (http://links.lww.com/JBJS/I38).
